# In vivo targeting of protein antigens to dendritic cells using anti‐DEC‐205 single chain antibody improves HIV Gag specific CD4^+^ T cell responses protecting from airway challenge with recombinant vaccinia‐gag virus

**DOI:** 10.1002/iid3.151

**Published:** 2017-03-13

**Authors:** Loveline N. Ngu, Nadesh N. Nji, Georgia E. Ambada, Bertrand Sagnia, Carol Ngane Sake, Jules Colinc Tchadji, Ghislain Donald Njambe Priso, Abel Lissom, Thibau Flaurant Tchouangueu, Denis Manga Tebit, Alain Bopda Waffo, Chae Gyu Park, Ralph M. Steinman, Klaus Überla, Godwin W. Nchinda

**Affiliations:** ^1^ Department of Biochemistry University of Yaounde Yaounde Cameroon; ^2^ Laboratory of Vaccinology/Biobanking of The Chantal Biya International Reference Center for Research on The Prevention and Management of HIV/AIDS Yaounde Cameroon; ^3^ Department of Animal Biology and Physiology University of Yaoundeone Yaounde Cameroon; ^4^ Department of Microbiology University of Yaoundeone Yaounde Cameroon; ^5^ Department of Biochemistry University of Dschang Cameroon; ^6^ Myles Thaler Center for AIDS and Human Retrovirus Research Department of Microbiology Immunology and Cancer Biology Charlottesville Virginia USA; ^7^ Department of Biological Sciences # 223 Alabama State University Montgomery Alabama USA; ^8^ Laboratory of Immunology, Severance Biomedical Science Institute, Brain Korea 21 PLUS Project for Medical Science Yonsei University College of Medicine Seoul Republic of Korea; ^9^ Laboratory of Cellular Physiology and Immunology and Chris Browne Center for Immunology and Immune Diseases Rockefeller University New York New York USA; ^10^ University Hospital Erlangen Institute of Clinical and Molecular Virology Erlangen Germany

**Keywords:** Antigens, dendritic cells, immunization

## Abstract

**Introduction:**

Targeting antigens to dendritic cells (DCs) in vivo via a DC‐restricted endocytic receptor, DEC205, has been validated to enhance immunity in several vaccine platforms. Particularly atttractive is selected delivery of proteins to DCs in vivo because it enables proteins to be more immunogenic and provides a cheaper and effective way for repeated immunizations.

**Methods:**

In this study, we tested the efficacy of a single chain antibody to DEC205 (scDEC) to deliver protein antigens selectively to DCs in vivo and to induce protective immunity.

**Results:**

In comparison to soluble Ovalbumin (OVA) antigen, when recombinant scDEC:OVA protein was injected subcutaneously (s.c.) into mice, the OVA protein was selectively presented by DCs to both TCR transgenic CD8^+^ and CD4^+^ T cells approximately 500 and 100 times more efficient than soluble OVA, respectively, and could persist for seven days following s.c. injection of the scDEC205:OVA. Similarly selective targeting of HIV Gag P24 to DCs in vivo using scDEC‐Gag protein plus polyICLC vaccine resulted in strong, long lasting, polyfuntional CD4^+^ T cells in mice which were protective against airway challenge by a recombinant vaccinia‐gag virus.

**Conclusion:**

Thus targeting protein antigens to DCs using scDEC can be used either alone or in combination with other strategies for effective immunization.

## Introduction

Recombinant protein vaccines are attractive because they enable immune responses to be focused on protective antigens. Due to their potent ability to prime immune responses, dendritic cells (DCs), loaded with antigen in vitro and in vivo, are presently explored as vaccine candidates against cancer and infectious diseases (reviewed in 1, 2, 3, 4, 5, 6, 7, 8, 9, 10). Our group and others have previously demonstrated that selective delivery of proteins to DCs in vivo allowed proteins to be more immunogenic and provided a cheaper and effective way for repeated immunizations 11, 12, 13, 14, 15, 16, 17, 18, 19, 20, 21, 22, 23. Our strategy involving targeting antigens to DCs via antigens coupled to a monoclonal antibody against a DC‐restricted endocytic receptor, DEC205, led to efficient immunity 12, 13, 14, 23, 24, 25, 26, 27, 28, 29, 30, 31, 32, 33. Here we describe a simple and cheap approach of targeting whole protein antigen to DCs in vivo, based on a single chain antibody to DEC205 (scDEC) fused to a protein antigen of interest. Due to its small size scDEC offers several attractive capacities including rapid tissue penetration, low cost of production and no Fc domain. The absence of an Fc domain eliminates potential interaction with Fc receptor‐expressing cells thereby improving not only specific targeting to DCs but also allowing for repeated immunizations without inducing deleterious host antibody responses 34, 35, 36, 37. In prior studies our group and others have demonstrated the immunogenicity of scDEC‐targeted antigen after incorporation in both DNA and viral vectored vaccines 25, 31, 38, 39. Here we prioritize scDEC‐protein vaccines because they offer a unique opportunity for scaling up strategies optimized with the parental anti‐DEC205 monoclonal antibody (αDECmAb)‐protein vaccines for eventual application in underdeveloped countries. Since scDEC might improve pharmacokinetics by eliminating rapid clearance of immunogen by Fc receptors, there is need to compare scDEC and αDECmAb to determine vaccine delivery efficiencies. We found out that small amounts of purified scDEC‐OVA, when injected subcutaneously (s.c.) into mice, could target OVA efficiently to DCs leading to both MHC class I and II presentation in vivo. In comparison to soluble OVA, injection of scDEC‐OVA protein increased in vivo presentation of antigenic peptides to MHC‐I and MHC‐II‐restricted T‐cells by at least a factor of 250 and 20, respectively. This presentation persisted for seven days as against 15 days when mice were similarly treated with the parental αDECmAb‐OVA protein. On the other hand s.c. injection of scDEC‐Gag in combination with polyICLC resulted in robust HIV Gag specific polyfunctional CD4^+^ T Cells which were protective to airway challenge by recombinant vaccinia‐gag virus. As such scDEC‐protein vaccines can be used either alone or in combination with other strategies for effective immunization.

## Materials and Methods

### Cell lines, media, and antibodies

The generation of CHO‐neo and CHO‐mDEC‐205 cell lines was described previously 13. Antibodies to TCR specificities (Vß_5.1/5.2_/MR9‐4; Vα_2_/20.1) and other cell surface markers CD11c, CD8α, and CD4 were purchased from BD Biosciences. Magnetic microbeads were from Miltenyi Biotec. Other antibodies included rabbit αOvalbumin (Chemicon, EMD Millipore, USA), mouse anti‐c‐Myc, mouse anti‐GFP, goat antirabbit‐FITC and goat antimouse‐FITC (Biosource International, Inc, USA), and KC57FITC (FITC‐αP24, Coulter).

### Vector construction

All vectors generated and used in this work have previously been described in Ref. 30.

### scDEC‐fusion protein purification from the supernatants of stably transfected CHO cells

The scDEC‐OVA, scCont‐OVA, scDEC‐Gag, and scCont‐Gag fusion proteins were purified from the supernatants of stably transfected CHO cells using Ni‐NTA agarose (Qiagen, Valencia, USA) through batch purification protocol according to the manufacturer's instructions. The purified proteins were concentrated by spin columns (EMD Millipore, MA, USA) and dialysed overnight against PBS. Protein concentration was estimated using the BCA Protein Assay Kit according to manufacturer's instructions (Lifetechnology, USA).

### SDS–PAGE and immunoblot analysis

The purified supernatants were resolved on 10% SDS–PAGE under reducing conditions and either directly Coomassie stained or blotted onto nitrocellulos (Amersham) by standard techniques. The blotted protein was blocked with Roti‐Block (Rott) and then reacted with either rabbit anti‐ovalbumin polyclonal antibody (1:10000, Chemicon) followed by anti‐rabbit peroxidase conjugate (1:2000). Bands were visualized with ECL substrate (Amersham). For the detection of HIV Gag p24 purified protein was resolved on a 10% SDS–PAGE and treated similarly except that that HRP‐conjugated anti‐p24 mAb was used for direct detection after blotting.

### Mice

C57BL/6(B6), TAP^−/−^, DEK^−/−^, OT‐I, OT‐II, and the CD45.1^+^ OT‐I mice were obtained from Charles Rivers and maintained under standard conditions in the Rockefeller University Animal Facility. All animals were used between 6 and 10 weeks under the guidelines of the Rockefeller University Institutional Animal Care and Use Committee. All necessary ethical approvals has been also acquired from the participating institutions.

### In vivo capture of scDEC205‐OVA fusion protein

B6 mice were injected subcutaneously in the four paws with 5 μg of scDEC‐OVA fusion proteins. Lymph nodes (popliteal, inguinal, axillary, branchial mesenteric) and spleen were harvested 24 h later and DCs isolated. Briefly single cell suspensions were prepared with 400 U/ml collagenase (Roche) for 25 min at 37°C. The cells were then incubated with anti‐mouse CD11c microbeads for 30 min at 37°C. CD11c^+^ (DC‐enriched) and negative cells were separated by application of a magnetic field. CD8^+^ or CD4^+^ T cells were prepared from OT‐I or OT‐II mice, respectively. OT‐I and OT‐II T cells were purified from single cell suspensions of lymph node or spleen cells by negative selection using hybridoma supernatants directed against MHC‐II, F4/80, B220, NK1.1, and CD4 or CD8 and goat anti‐rat Dynabeads® (Dynal, Thermo Fisher Scientific, USA) at a ratio of 4 beads to 1 target cell. Culture medium was RPMI, 7% FBS, 100 U/ml penicillin streptomycin mixture, 0.25 mg/ml fungizone, 10 mM Hepes, and 55 μM ß‐mercaptoethanol.

### Labeling of cells with CFSE for in vivo T cells proliferation responses

CD8^+^ or CD4^+^ T cells were prepared from OT‐I or OT‐II mice respectively. OT‐I and OT‐II T cells were purified from single cell suspensions of lymph node or spleen cells by negative selection using hybridoma supernatants directed against MHC‐II, F4/80, B220, NK1.1, and CD4 or CD8 and goat anti‐rat Dynabeads® (Dynal) at a ratio of 4 beads to 1 target cell. OT‐I and OT‐II cells at 10^7^ cells/ml were incubated with CFSE (Molecular probes; 5 μM) for 10 min at 37°C. An equal volume of FCS was added, and the cells washed two times with PBS/0.1% BSA and twice with PBS. 2 × 10^6^ labelled OT‐I and 3 × 10^6^ labelled OT‐II cells were injected intravenously into B6 recipients respectively. 24 h later scfv:OVA fusion proteins were injected into all 4 paws. After 3 days, both draining lymph nodes and spleen cells were stained separately for Vß_5.1/5.2_, CD8 (OT‐I), Vα_2_, CD4 (OT‐II) and evaluated by multicolour flow cytometry.

### Flow cytometry

Multicolor flow cytometry was used to monitor in vivo functional responses by analysing the proliferation of carboxyflourescein diacetate succinimidyl ester (CFSE)‐labelled T cells as assed by progressive halving of the amount of flourescein per cell. We used a FACScalibur™ (BD Biosciences, USA) with subsequent analysis of data in CELLQuest™ (BD Biosciences) or Flowjo^R^ (Tree Star, OR, USA). Multiparametric flowcytometry for HIV Gag specific T cell responses was carried out as previously reported in our group 25, 30, 31.

### In vitro presentation of OVA by DCs

B6 mice were injected with 5 ug of scDEC205: OVA fusion proteins subcutaneously in the 4 paws. Lymph nodes (popliteal, inguinal, axillary, branchial mesenteric) and spleen were harvested 24 h later and DCs isolated. Briefly single cell suspensions were prepared with 400 U/ml collagenase (Roche) for 25 min at 37°C. The cells were then incubated with anti‐mouse CD11c microbeads for 30 min at 37°C. CD11c^+^ (DC‐enriched) and negative cells were separated by application of a magnetic field. CD8^+^ or CD4^+^ T cells were prepared from OT‐I or OT‐II mice respectively as described previously. Culture medium was RPMI, 7% FBS, 100 U/ml penicillin streptomycin mixture, 0.25 mg/ml fungizone, 10 mM Hepes, and 55 uM ß‐mercaptoethanol.

### Assays for HIV‐specific immune T cells

Female C57/B6 mice were injected twice i.p. at 4 weeks interval with scDEC‐ or scCont‐fusion protein together with polyICLC (50 µg, oncovir) as adjuvant. Fourteen days following the last vaccination spleens were dissociated and HIV gag‐specific CD3^+^ CD4^+^ splenic T cells were analyzed for simultaneous production of IFNγ, IL‐2, and TNF‐α. To determine the breadth of HIV Gag‐specific T cell responses, splenocytes were restimulated in vitro with peptides mix spanning the entire Gag p24 protein 25, 26, 29, 30, 31 or a negative unreactive control peptide mix consisting of HIV Gag p17 pool 1 or HA in the presence of 2 ug/ml of anti‐CD28 (clone 37.51) for 6 h, adding 10 μg/ml brefeldin A (Sigma–Aldrich) for the last 4 h to accumulate intracellular cytokines. Dead cells were excluded using live/dead fixable dead stain kit (Aqua live/dead; Thermo Fischer Scientific). After blocking FcyR receptors, the cells were stained with antibodies to CD3‐pacific blue, CD4‐percp, CD8‐alexa‐750, and Aqua live/dead stain for 20 min at 37°C. Cells were washed, fixed (Cytofix/cytoperm; BD Bioscience), permeabilized with Permwash and stained with antibodies to IFNγ (IFNγ‐alexa‐700), IL‐2 (IL‐2‐FITC), and TNF‐α (TNF‐α‐PE‐CY7) for 15 min at room temperature. All antibodies were from Ebioscience. We use BD LSRII for acquisition and data analysis was with Flowjo (Tree Star).

### Vaccinia‐gag protection assay

Nembutal‐anesthetized mice were challenged with 10^5^ PFU/mouse of infectious recombinant vaccinia‐gag virus by the intranasal route, in 35 μl PBS with Mg/Ca. A negative control was vaccinia‐OVA virus. The weight of each animal (groups of 5) was determined daily for 6 days following challenge. Then mice were euthanized, the lungs were harvested and homogenized in transport medium (0.1% gelatin in PBS), and stored in duplicates at −80°C prior to virus titration. Lung virus titers of individual mice in each group were determined by plaque assay on monolayers of CV‐1 cells as described 24, 25, 29, 30, 31.

### Statistics

Postchallenge mean vaccinia lung virus titers and mean percentage in weight loss were compared between vaccination groups using one tailed Student's *t* test. Differences were considered significant at *P* < 0.05 after analysis using prism 6 Graph Pad Software.

## Results

### In vivo targeting and presentation of antigen to CD8^+^ T cells using scDEC‐OVA

**Figure 1:**
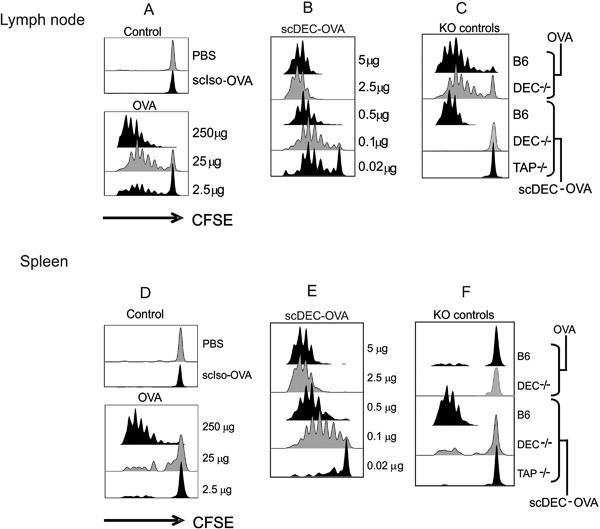
Antigen presentation to OT‐I T cells following scDEC targeting in vivo. scDEC‐OVA induces stronger in vivo proliferation of OT‐I T cells than OVA alone. C57BL/B6 mice were injected intravenously with 2 × 10^6^ CFSE‐labelled OT‐I T‐cells then graded doses of scDEC‐OVA (A) or OVA (B) subcutaneously 24 h later. Three days after scDEC injection, draining lymph node cells were harvested and the expansion of CD8^+^ Vα_2_ß_5.1/5.2_ evaluated by flow cytometry for CFSE dilution. scDEC‐OVA elicited better presentation of OVA derived peptide than the scCon‐OVA and soluble OVA. In contrast to C57BL/B6 (B6) presentation of peptide from scDEC‐OVA could not be seen in DEC205 K (DEC^−/−^) and TAP knock out (TAP^−/−^) mice. On the contrary OVA peptide from B6 and DEC^−/−^ could be presented (C). In (D) to (F) similar data is shown for the spleen. Here as shown in the KO controls presentation was less efficient in the spleen (F). The experiment was repeated several times with similar results.

**Figure 2:**
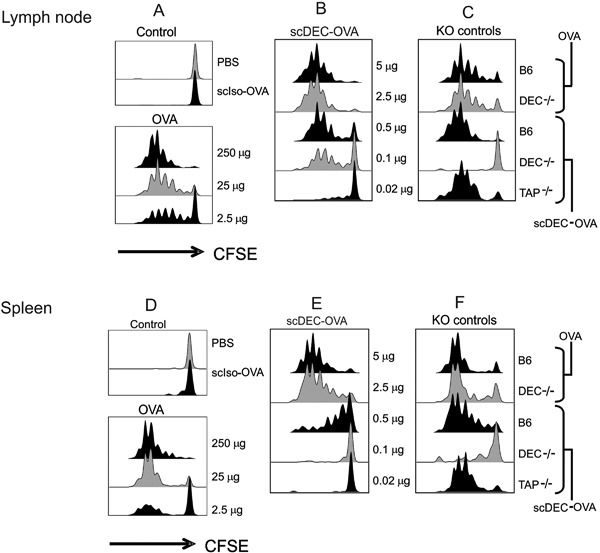
Antigen presentation to OT‐II T cells following scDEC targeting in vivo.scDEC‐OVA induces stronger in vivo proliferation of OT‐I I T cells than OVA alone. C57BL/B6 mice were injected intravenously with 3 × 10^6^ CFSE‐labelled OT‐II T‐cells then graded doses of scDEC‐OVA (A) or OVA (B) subcutaneously 24 h later. Three days after scDEC injection, draining lymph node cells were harvested and the expansion of CD4^+^ Vα_2_/20.1 evaluated by flow cytometry for CFSE dilution. scDEC‐OVA elicited better presentation of OVA derived peptide than the scCont‐OVA and soluble OVA in both the lymph node (B) and spleen (E). On the other hand presentaion was abrogated in DEC^−/−^ mice in both the lymph nodes and spleen (compare C and F). In contrast to Figure 2 OT‐II presentation of peptide from scDEC‐OVA could be seen in both TAP knock out (TAP^−/−^) and B6 mice but not in DEC205 (DEC^−/−^). On the contrary OVA peptide form B6 and DEC^−/−^ mice was well presented. The experiment was repeated several times with similar results.

The parental αDECmAb has previously been demonstrated to target protein efficiently to DCs leading to efficient presentation of antigens to both MHC classes I and II pathways 11, 12, 33. To verify that DCs targeted with the scDEC‐OVA could present antigens in situ, we performed adoptive transfer of OVA‐specific TCR transgenic T cells (CD8^+^ MHC class I‐restricted OT‐I) labeled with CFSE one day before the injection of scDEC‐OVA, scCont‐OVA, or soluble OVA. After 3 days, the draining lymph nodes (Fig. 1A–C) and spleen (Fig. 1D–F) were evaluated for T cell proliferation as assayed by CFSE dilution. Virtually all of the OT‐I cells in the draining lymph nodes and spleen entered cell cycle and underwent several rounds of cell divisions with a dose of just 100 ng of the scDEC‐OVA (Fig. 1B and E). In contrast, soluble OVA required the dose containing approximately 500‐fold more protein (250 ug OVA versus 0.5 ug scDEC‐OVA) to induce comparable proliferative responses and again scCont‐OVA elicited little or no proliferation (Fig. 1A and D). To determine whether MHC class I presentation by scDEC‐OVA was TAP dependent, we performed similar experiments with TAP‐deficient mice (TAP^−/−^, Fig. 1C and F). In the absence of TAP, proteasome‐processed peptides in DCs apparently failed to move into the endoplasmic reticulum for association with MHC class I after uptake of scDEC‐OVA, but the same cells were able to present OVA on MHC class II (compare Fig. 1C and E with Fig. 2C and E). To prove that DEC‐205 but not Fcγ receptors were mediating uptake and presentation of antigens, we verified that CFSE dilution was abolished when similar experiments were performed in DEC‐205 deficient mice (DEC‐205^−/−^, Fig. 1C and F).

### In vivo targeting and presentation of antigen to CD4^+^ T cells using scDEC‐OVA

Similar adoptive transfer of CD4^+^ MHC class II‐restricted OT‐II Transgenic T cells followed by subsequent injection of scDEC‐OVA revealed strong presentation of antigen to OT‐II T‐cells (Fig. 2). Thus recombinant scDEC‐OVA efficiently targets antigens to DCs leading to presentation by both MHC class I and MHC class II pathways in vivo. Briefly mice were similarly treated as described above then 3 days later, the draining lymph nodes (Fig. 2A–C) and spleen (Fig. 2D–F) were evaluated for T cell proliferation as assayed by CFSE dilution. Most but not all of the OT‐II cells in the draining lymph nodes (Fig. 2A–C) and spleen (Fig. 2D–F) entered cell cycle and underwent several rounds of cell divisions. However, for OT‐II cells, a dose of at least 100 ng of scDEC‐OVA was necessary to initiate proliferation in the draining lymph nodes (Fig. 2B). The minimal dose required for scDEC‐OVA presentation to OT‐II cells in the spleen was much higher, that is at least 500 ng (Fig. 2E). For soluble OVA, approximately 100 fold more protein (250 ug OVA versus 2.5 ug scDEC‐OVA) was required to induce comparable responses of OT‐II T cell proliferation and, again, scCont‐OVA elicited little or no proliferation (Fig. 2A and D). Since MHC class II presentation is independent of TAP when similar experiments were performed in TAP‐deficient mice (TAP^−/−^, Fig. 2C and F) OVA presentation to OT‐II T cells was not abrogated. To prove that DEC205 but not Fcγ receptors were mediating OT‐II presentation, we verified that CFSE dilution was abolished when similar experiments were performed in DEC205 deficient mice (DEC‐205^−/−^, Fig. 2C and F).

### Comparison between αDECmAb‐OVA and scDEC‐OVA

**Figure 3:**
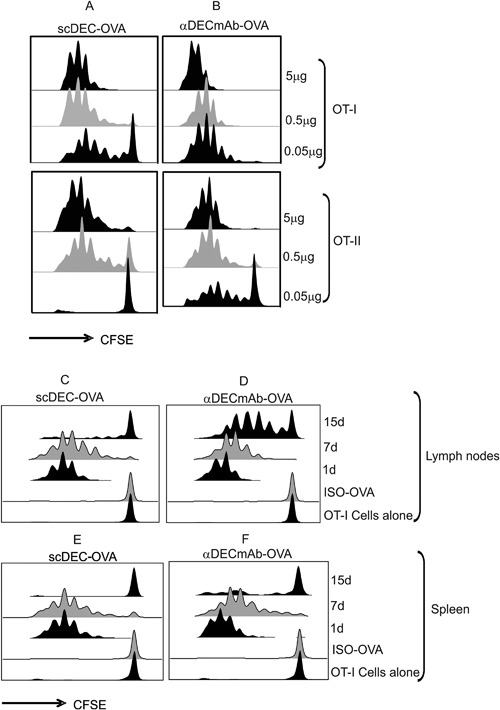
Comparison of Antigen presentation to T cells following scDEC‐OVA and αDECmAb‐OVA targeting in situ. B6 mice were injected intravenously with 2 × 10^6^ or 3 × 10^6^ CFSE‐labeled OT‐I and OT‐II T‐cells respectively 24 h prior to the s.c. injection of graded doses of scDEC‐OVA and αDECmAb‐OVA as depicted. Three days after protein injection draining lymph nodes cells were harvested and the expansion of CD8^+^ Vα_2_ß_5.1/5.2_ (A) or CD4^+^ Vα_2_/20.1 (B) T cells evaluated by flow cytometry. B6 mice were injected s.c with 5 ug of scDEC‐OVA or αDECmAb‐OVA or the isotype control and then evaluated for peptide presentation 1, 7, or 15 days later. Treated mice were injected intravenously with 2 × 10^6^ or 3 × 10^6^ CFSE‐labeled OT‐I and OT‐II T‐cells respectively. Three days later draining lymph nodes cells were harvested and the expansion of CD8^+^ Vα_2_ß_5.1/5.2_ (C) or CD4^+^ Vα_2_/20.1 (D) T cells evaluated by flow cytometry. For mice targeted with scDEC‐OVA presentation to OT‐I T cells persisted for up 7 days as compared to 15 days for αDECmAb‐OVA targeted mice (A). Similar data for the expansion of OT‐1 T cells in the spleen is shown in (E) for scDEC‐OVA and (F) for aDECmAb‐OVA.

When compared to OVA fused to recombinant αDECmAb (parental antibody), targeting of DEC205 by the single chain scDEC‐OVA was comparable to the recombinant αDECmAb‐OVA except that higher doses (>50 ng) of scDEC‐OVA were required (Fig. 3 compare A and B). At doses less than 100 ng, scDEC‐OVA was comparatively less efficient. Following s.c. injection of scDEC‐OVA, the antigen presentation to TCR transgenic CD8^+^ T cells persisted for up to 7 days for the scDEC targeted proteins as compared to 15 days for the recombinant αDECmAb‐OVA (compare Fig. 3C and E with D and F). The αDEC monoclonal antibody (αDECmAb) has previously been demonstrated to efficiently target antigens to CD11c^+^ DCs 11, 33, 34. To determine whether DCs could actually be targeted by recombinant scDEC‐OVA protein in vitro, CD11c^+^ and CD11c^−^ cells were isolated from the draining lymph nodes and spleen of naïve mice and co‐cultured with OT‐I T‐cells in 1 μg/ml of scDEC‐OVA before measuring the proliferation of T cells by uptake of [3H] thymidine at 48–78 h. We detected strong presentation by the CD11c^+^ and not by the CD11c^−^ cells (Fig. S1A). To verify that the scDEC‐OVA targeted DCs would be responsible for the antigen presentation in vivo, we next isolated CD11C^+^ and CD11C^−^ cells from mice injected subcutaneously with the scDEC‐OVA or αDECmAb‐OVA as positive control (data not shown). When assayed for antigen presentation to OT‐I T cells in vitro at 24 h after injection of scDEC‐OVA, we could detect antigen presentation efficiently by the CD11C^+^ but not by the CD11C^−^ cells (Fig. S1B). Therefore the scDEC‐OVA could efficiently target OVA to DCs in vitro and in vivo leading to antigen presentation.

### scDEC‐Gag protein vaccine in combination with polyICLC induces robust durable polyfunctional Gag specific CD4^+^ T cell responses

**Figure 4:**
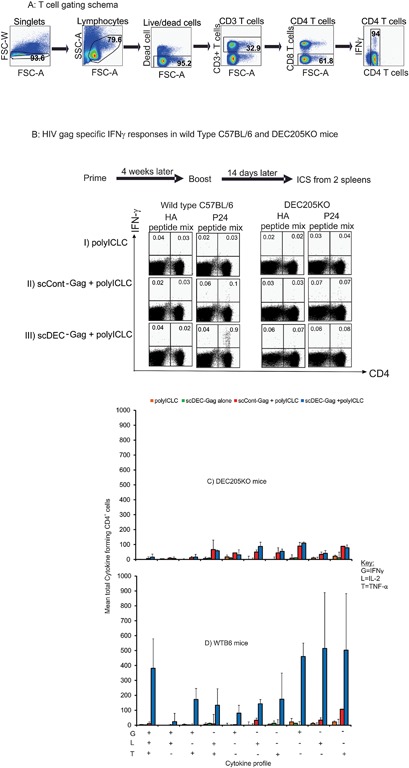
scDEC‐Gag plus polyICLC vaccination results to robust polyfunctional CD4^+^ T cell responses. C57BL/6 mice were immunized twice s.c. at 4 weeks interval as indicated on the Y‐axis 14 after the last vaccination bulk splenocytes were assessed for HIV Gag specific T cell immunity. Splenocytes were restimulated either with unreactive peptides or with an HIV Gag p24 peptide mix then IFN‐γ, IL‐2, and TNF‐α production in response to peptide was evaluated by intracellular cytokine staining 6 h later in T cell (B). In Figure 4A data is shown for the gating schema of the cytokine forming cells while in (B) is dot plot is shown for IFN‐γ production by CD4^+^ T cells in both wild type B6 and DEC205KO mice. Polyfunctional Gag specific CD4^+^ T cell responses 14 days after the last boost in DKO mice (C) or wildtype C57BL/6 mice (D). All data are mean ± SD of three repeat experiments involving five female C57BL/6 mice per group.

Effective HIV Gag specific T cell responses are vital in the control of viremia and could be a useful component of therapeutic or prophylactic vaccines against HIV‐1 infection. We have shown in previous reports that vaccination with αDECmAb‐Gag in combination with polyICLC resulted in strong polyfunctional Gag specific CD4^+^ T cell responses 14, 25, 30, 34. Similarly, scDEC‐Gag alongside its control scCont‐Gag fusion proteins (Supplementary Fig. S1) were expressed purified and characterized as previously described 29, 30, 31, 33. To assess the immunogenicity of scDEC‐Gag plus polyICLC vaccine we immunized C57BL/6 mice twice at 4 weeks interval as previously reported 14, 25, 30, 36 with the targeted vaccine as well as the control. Fourteen days after the last vaccination splenocytes were assessed for Gag specific T cell responses by multi‐parametric flow cytometry as previously described 24, 25, 29, 30, 31. As shown in Figure 4 relative to the control vaccine scDEC‐Gag plus polyICLC vaccine (compare Fig. 4B rows III and IV) induced significantly higher Gag specific CD4^+^ T cells immune responses (*P* < 0.05) which were seven fold stronger than the control group. This response was DEC205 dependent as a similar in DEC205 knock out mice (DKO) showed no gag specific immune responses.The targeted vaccine also resulted in superior numbers of single CD4^+^ T cells simultaneously expressing multiple cytokines including IL‐2, IFN‐γ, and TNF‐α. In three repeat experiments scDEC‐Gag plus polyICLC resulted in superior polyfunctional CD4^+^ T cells which were significantly higher than the scCont‐Gag (*P* < 0.001) (Fig. 4C). When DKO mice were similarly vaccinated little or no Gag specific CD4^+^ T cells were induced clearly indicating that DEC‐205 targeting was implicated in the enhanced immunogenicity of the targeted vaccine (compare Fig. 4C with D). Supplementary Figure S3 shows data for DEC205 expression in the vaccinated mice. These data show that in the absence of DEC205 there was no difference in the immunogenicity of scDEC‐Gag and scCont‐Gag. Again as previously reported after vaccination with αDECmAb‐Gag protein plus polyIClC 25, 30 no detectable Gag specific CD8^+^ T cells were observed following a similar treatment with scDEC‐Gag protein in combination with polyICLC.

### Comparing the immunogenicity of parental αDECmAb‐Gag plus polyICLC with scDEC‐Gag plus polyICLC

**Figure 5:**
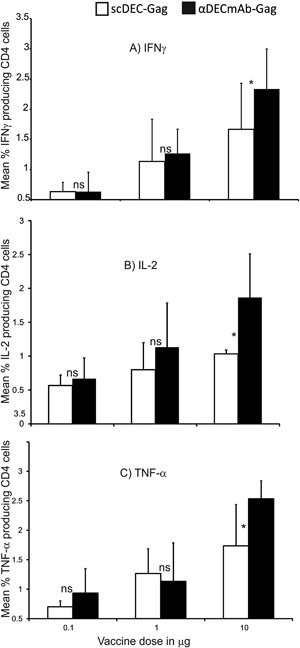
Comparing the immunogenicity of parental αDECmAb‐Gag plus polyICLC with scDEC‐Gag plus polyICLC. Groups of five female 6–10 weeks C57BL/6 mice vaccinated twice 4 weeks apart either with graded doses of scDEC‐Gag or αDECmAb‐Gag in combination with 50 μg polyIC as indicated on the X‐axis. T cells responses were monitored 1 week after the boost. (A) mean percentage IFNγ producing CD4^+^ T cells (*P = 0.32; *t*‐test). (B) as in (A) but shows IL‐2 production (*P = 0.09; *t*‐test). (C) as in (A) but showing TNF‐α producing CD4 T cells 7 days post boost (*P = 0.14; *t*‐test). Data represents mean ± SD of 3 three repeat experiments with five mice per group per experiment.

As at least 100 ng of scDEC‐OVA was needed to produce the same effect like αDECmAb‐OVA we immunize C57BL/6 mice with graded doses of the HIV gag fusion protein vaccines as indicated the X‐axis of Figure 5A–C). Each dose of vaccine was given with 50 μg of plyICLC as described in materials and methods. Four weeks after priming the mice received a second dose of the same treatment. Seven days later HIV gag specific T cells response are determined as described above. In Figure 5 data is shown for the mean percentage cytokine production by CD4 T cells following in vitro restimulation with HIV gag P24 restrictive peptides. There was no significant difference in the mean percentage cytokine production for both vaccines after vaccination with 0.1 and 1 μg protein (compare Fig. 5A–C). However following vaccination with 10 μg protein; αDECmAb‐Gag protein in combination with polyICLC induce slightly higher but not significant levels (Student's *t* test) of IFNγ (Fig. 5A, *P* < 0.32), Il‐2 (Fig. 5B, *P* < 0.09), and TNF‐α (Fig. 5C, *P* < 0.14). Thus with respect to immunogenicity both vaccines induced similar T cell responsesin mice after s.c. immunization.

### scDEC‐Gag plus polyICLC vaccination elicits protection at a mucosal surface

**Figure 6:**
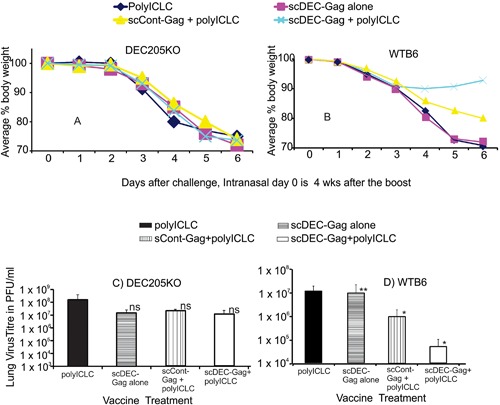
Dendritic cell targeted scDEC‐Gag protein vaccine results to protection in the airway. Groups of five female 6–10 weeks C57BL/6 mice vaccinated twice with 5 μg scDEC‐Gag with 50 μg polyIC as adjuvant 4 weeks apart. Twelve weeks after the boost, the mice received a lethal dose (10^5^ PFU) of recombinant vaccinia‐gag intranasally. Weight loss was monitored daily for 6 days after challenge for (A) DEC205KO or (B) wild type B6 mice, and vaccinia virus titres in the lung (PFU/lung) was measured after euthanizing at 6 days for (C) DEC205KO or (D)WTB6 mice. (mean ± SD of three experiments).

Twelve weeks following vaccination C57BL/B6 mice were challenged intranasally with recombinant vaccinia‐gag as previously described 9, 11, 12, 24, 25, 30, 31, 35. In Figure 6 data is shown for three long term repeat experiments. Control mice vaccinated with the empty scDEC were not protected relative to PBS‐injected mice, and they lost weight continuously during the challenge. Similarly DKO mice vaccinated and challenged as described above were not protected against weight loss (compare Fig. 6A and B). When virus titers in the lungs were assessed at day 6 post challenge scDEc‐Gag plus polyICLC vaccinated mice show up to 2.5 logs less viruses than the naïve mice. This lung virus titer was significantly less than mice (*P* < 0.05) vaccinated with scCont‐Gag plus polyICLC (compare Fig. 6C and D). On the other hand, similarly vaccinated and challenged DKO showed significantly higher lung virus titer (*P* < 0.05) in all groups clearly indicating the relevance of the DEC205 receptor in the targeted vaccination. Thus targeting protein using scDEC enables the induction of protective T cell immunity in the airway.

## Discussion

We showed that scDEC fusion protein is a simple method of targeting antigen to DC, leading to presentation both by the MHC I and II pathway. DEC205 targeting allows for selective loading of Ag unto DC which has been demonstrated to be the most potent APC, thus representing a useful strategy for increasing Ag presentation via both the MHC class I and II. We previously demonstrated that both viral vectored and DNA vaccines targeting the encoded antigen to DC in vivo improved T cell responses to gag 29, 30, 31, 32. Here we demonstrate the efficacy of scDEC‐fusion protein in improving HIV gag specific CD4^+^ T cell immunity in murine airwar through selective antigen targeting to dendritic cells in vivo, in combination with polyICLC as adjuvant.

The potential therapeutic use of scDEC fusion proteins has several advantages. The variable regions of antibodies comprise the smallest fragments containing a complete antibody binding site, and fusion molecules can be created without loss in binding function 29, 33, 34, 35, 37. Therefore, scDEC is an attractive tool for targeting whole protein Ag to DC in vivo. The fusion protein scDEC‐OVA and scDEC‐Gag with the small molecular size of the scDEC (38kda) is expected to be minimally immunogenic permitting repeated immunization without inducing deleterious anti‐host antibody responses. It can be produced in large amounts, in a short time and at a low cost, and it can be highly purified by immobilized metal affinity chromatography (IMAC), thus providing an ideal situation for large scale production.

Although the scDEC‐OVA as shown here was comparable to the αDECmAb‐antigen conjugate only at concentrations greater than 100 ng, it was approximately 500 fold more effective than soluble OVA. The requirement for large amounts of scDEC fusion proteins may not be a limitation for the induction effective immunity as a combination of scDEC‐Gag plus polyICLC resulted to similar CD4^+^ T cell responses like the parental antibody. As previously reported with the parental αDECmAb‐Gag conjugate scDEC‐gag resulted exclusively to CD4^+^ T cell responses which were qualitatively and quantitatively better than non‐targeted vaccination 9, 24, 25, 30, 34. Like previously reported by our group polyfuntional Gag specific CD4^+^ T cells were induced in a DEC205 dependent manner 30.

In summary, scDEC‐OVA and scDEC‐Gag fusion proteins have been developed that targets not only OVA effectively to CD11c^+^ cells (Supplementary Fig. S2) but also induced protective HIV specific T cell responses in the airway. This recombinant scDEC fusion protein efficiently targets DC both in vitro and in vivo thereby offering a simple approach for loading homogeneous Ag onto DC in vivo. Immunogenicity studies with a model HIV T cell vaccine also show that the scDEC‐Gag administered with polyICLC as an adjuvant enables protective HIV Gag specific T cells responses in the murine airway. Previous reports from our group had shown that this airway protection to recombinant vaccinia‐gag virus challenge was mediated by proliferative broad spectrum Gag specific CD4^+^ T cells responses 25, 30 following αDECmAb‐Gag vaccination. The induction of mucosal protective CD4^+^ T cells is particularly attractive to combat a challenging virus like HIV‐1 at its portal of entry 37. However there remains the also concern that activated CD4^+^ T cells at the portal of entry could present new targets for HIV‐1 infection. This may not be the case because we previously demonstrated that these CD4^+^ T cells would rather help the rapid induction of pathogen specific CD8^+^ T cells 26, thus this strategy can be exploited in combination with other vaccination approached to focus pathogen specific immune responses at the portal of entry.

## Supporting information

Additional supporting information may be found in the online version of this article at the publisher's web‐site.

**Figure S1**. Coomassie stain and FACS analysis of scDEC‐OVA/scDEC‐Gag binding to CHOmDEC205 expressing cells.

**Figure S2**. scDEC‐OVA targeted DCs processed and present antigen both in vitro and in vivo.

**Figure S3**. DEC205 expression in WT B6 mice and KO mice immunized with scDEC‐Gag plus polylCLC.

**Figure S4**. Durable HIV Gag specific CD4 T cell cytokine production after scDEC‐Gag plus polylCLC vaccination.
