# Supplementary figures and images for: In vivo targeting of protein antigens to dendritic cells using anti‐DEC‐205 single chain antibody improves HIV Gag specific CD4+ T cell responses protecting from airway challenge with recombinant vaccinia‐gag virus
Source: Immun Inflamm Dis. 2017 Mar 13;7(2):55–67. doi: 10.1002/iid3.151 (PMC6485703; doi:10.1002/iid3.151)

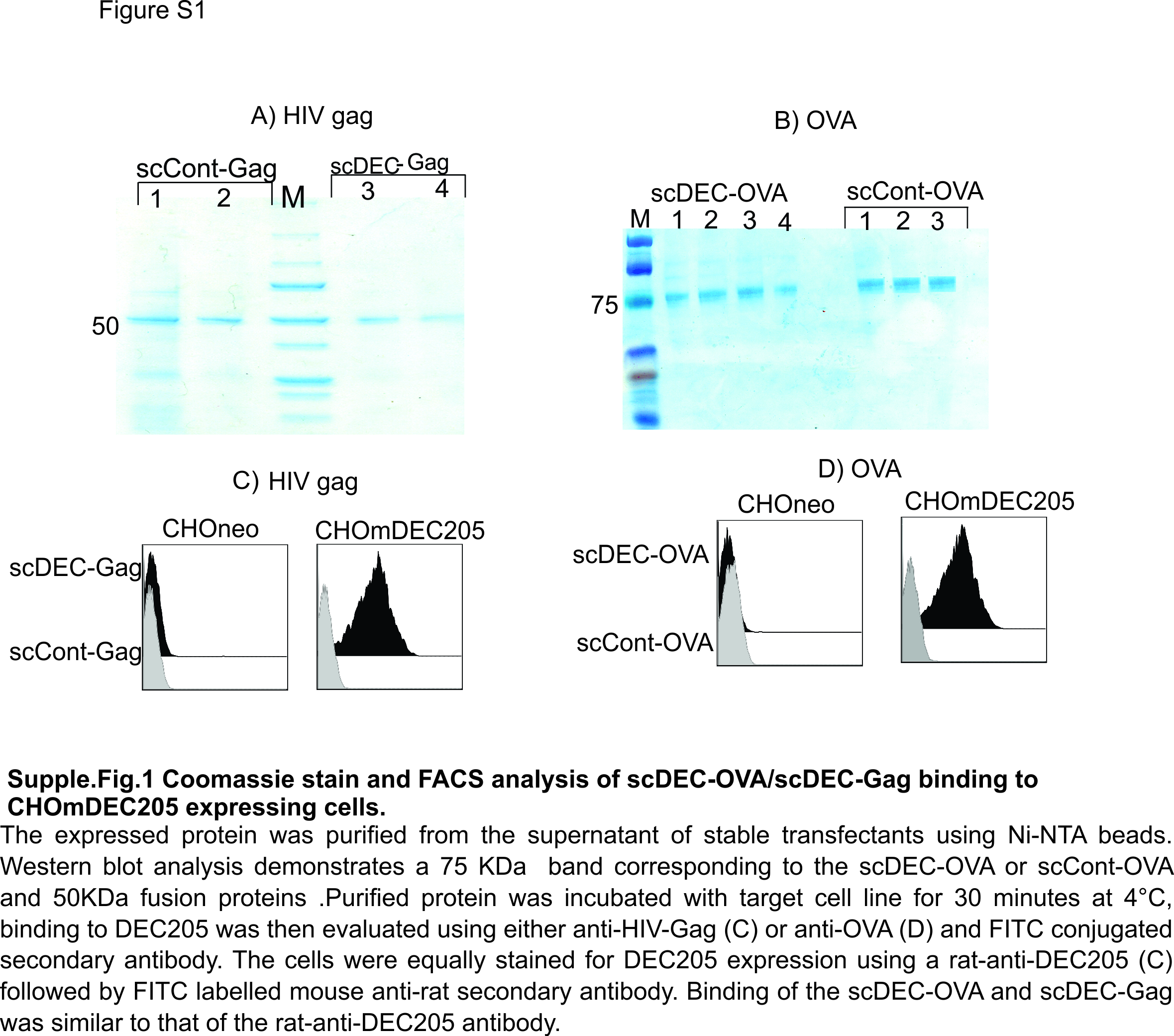

Supplement: Supplementary file 1 — Figure S1. Coomassie stain and FACS analysis of scDEC‐OVA/scDEC‐Gag binding to CHOmDEC205 expressing cells. [file IID3-7-55-s001.jpg]

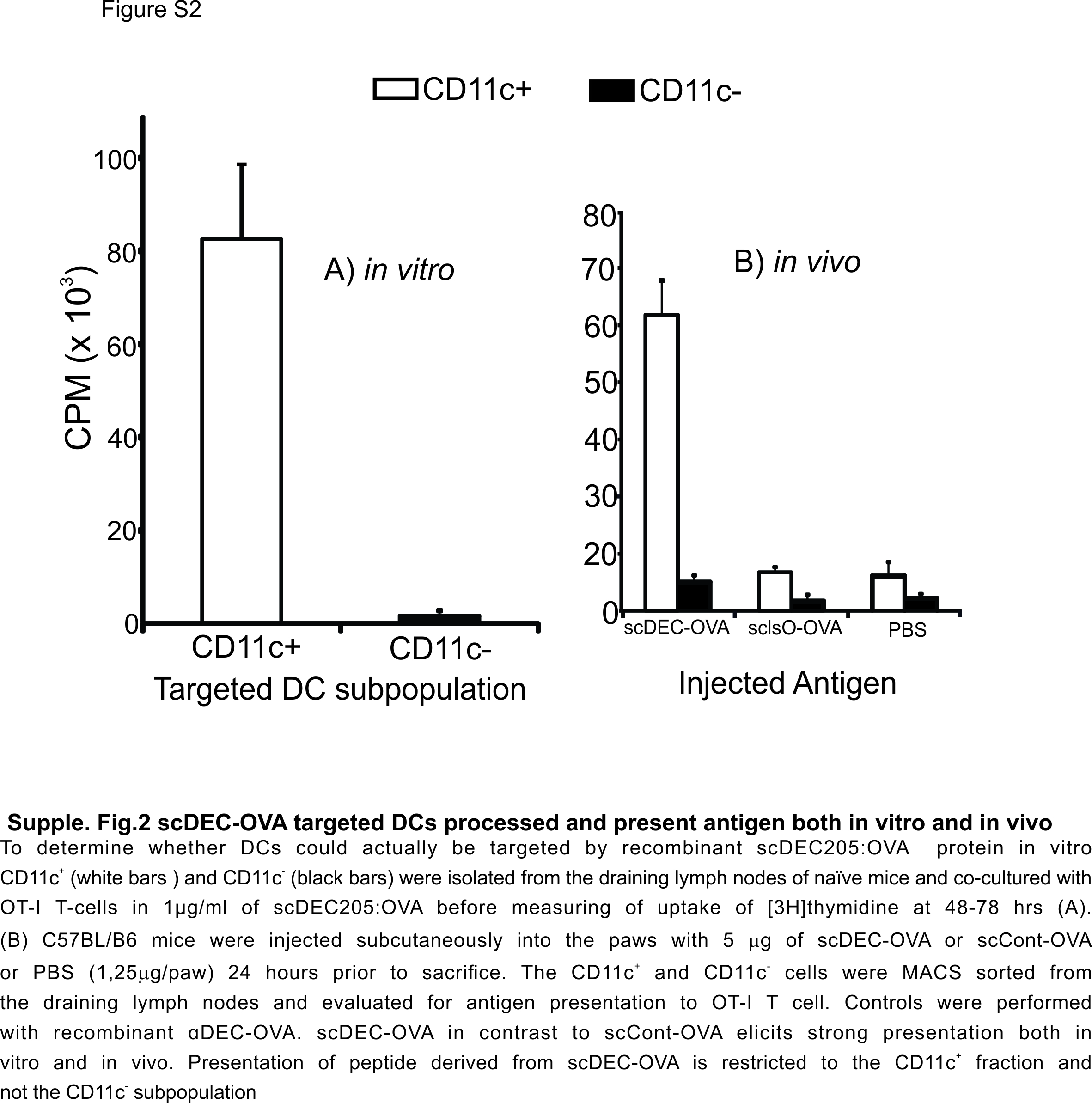

Supplement: Supplementary file 2 — Figure S2. scDEC‐OVA targeted DCs processed and present antigen both in vitro and in vivo. [file IID3-7-55-s002.jpg]

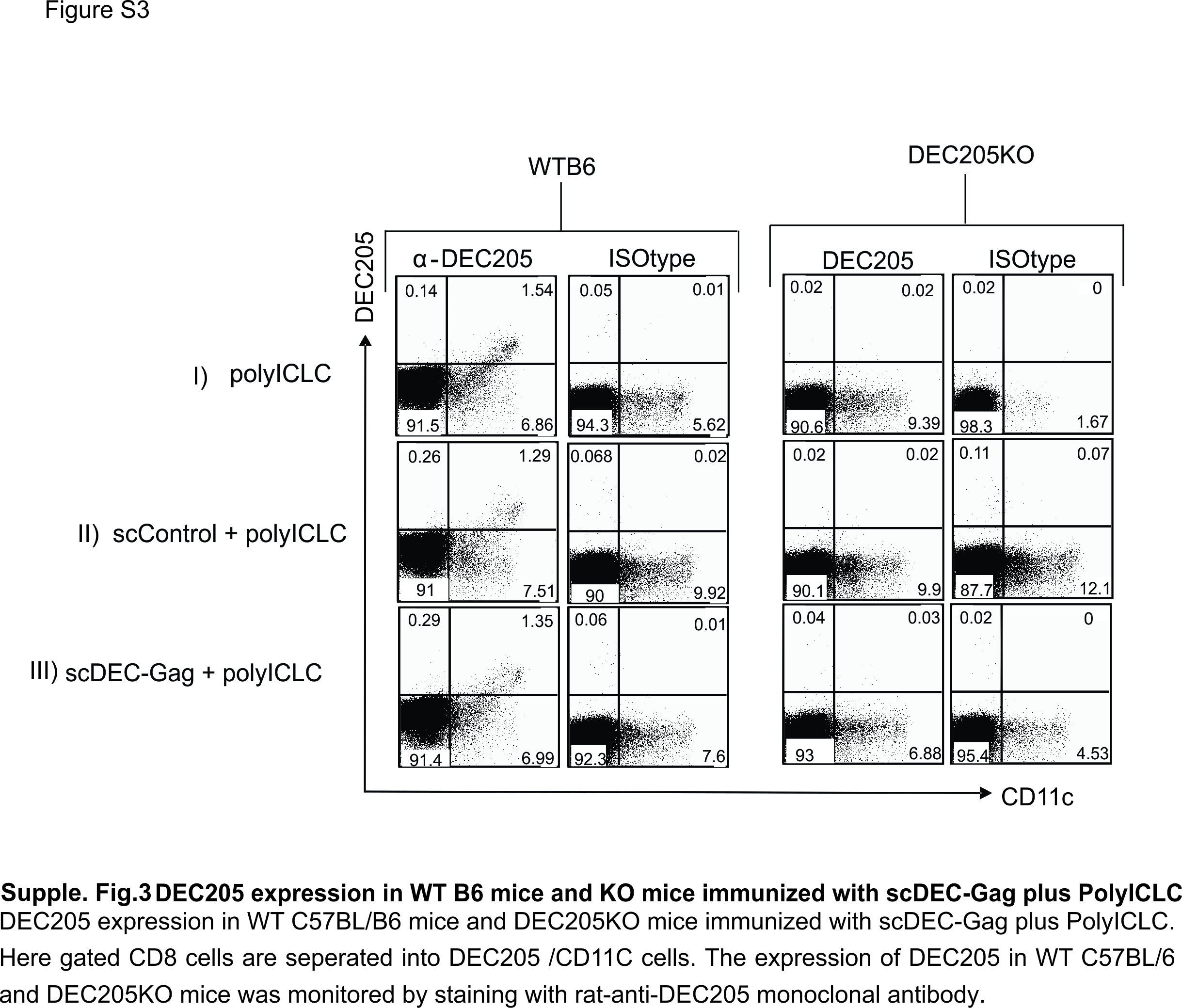

Supplement: Supplementary file 3 — Figure S3. DEC205 expression in WT B6 mice and KO mice immunized with scDEC‐Gag plus polylCLC. [file IID3-7-55-s003.jpg]

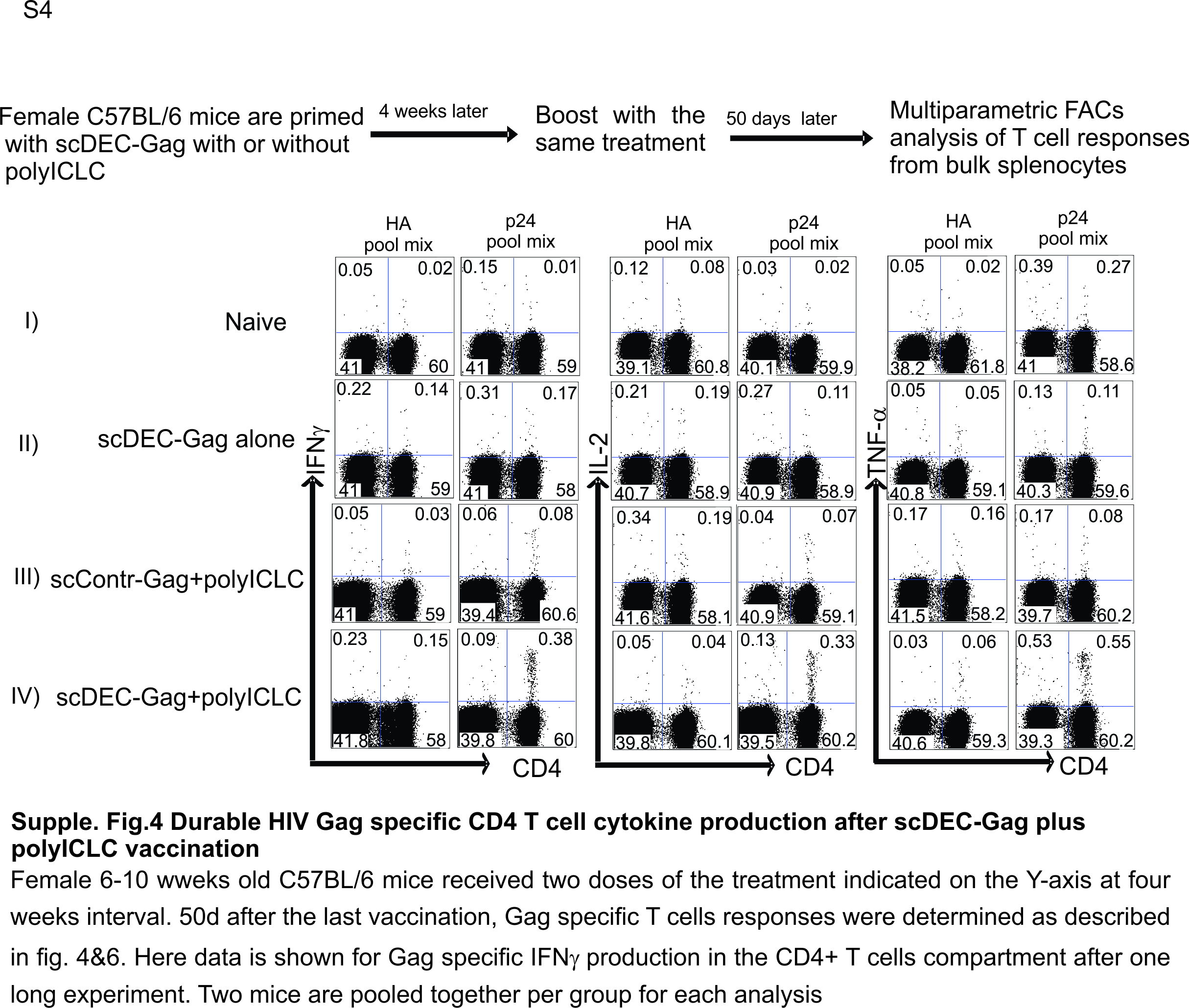

Supplement: Supplementary file 4 — Figure S4. Durable HIV Gag specific CD4 T cell cytokine production after scDEC‐Gag plus polylCLC vaccination. [file IID3-7-55-s004.jpg]
